# Molecular Analysis of the Differential Activity of Sox8 and Sox10 in Oligodendroglial Cells

**DOI:** 10.3390/ijms252413395

**Published:** 2024-12-13

**Authors:** Verena Dehm, Tim Aberle, Laura Guerrero Bladé, Jessica Aprato, Matthias Weider, Heinrich Sticht, Elisabeth Sock, Michael Wegner

**Affiliations:** 1 Institut für Biochemie, Friedrich-Alexander-Universität Erlangen-Nürnberg, D91054 Erlangen, Germany; verena.dehm@fau.de (V.D.); tim.aberle@fau.de (T.A.); jessica.aprato@fau.de (J.A.); heinrich.sticht@fau.de (H.S.); elisabeth.sock@fau.de (E.S.); 2 Zahnklinik3—Kieferorthopädie, Universitätsklinikum Erlangen, Friedrich-Alexander-Universität Erlangen-Nürnberg, D91054 Erlangen, Germany; matthias.weider@uk-erlangen.de

**Keywords:** Sox proteins, high-mobility-group domain proteins, transcription factor paralogs, myelin, glia, oligodendrocyte

## Abstract

Oligodendroglial cells generate myelin sheaths in the vertebrate central nervous system to render rapid saltatory conduction possible and express the highly related Sox8, Sox9 and Sox10 transcription factors. While Sox9 and Sox10 fulfill crucial regulatory roles, Sox8 has only a limited impact on oligodendroglial development and myelination. By replacing Sox10 with Sox8 or Sox9 in the oligodendroglial Oln93 cell line, and comparing the expression profiles, we show here that Sox8 regulates the same processes as Sox10 and Sox9, but exhibits a substantially lower transcriptional activity under standard culture conditions. Sox8 influences fewer genes in their expression and changes their expression level less drastically, despite comparable binding to relevant regulatory regions in oligodendroglial cells. Therefore, it is likely that Sox8 and Sox10 vary in their transcriptional activity because of differences in their interactions with partner proteins. Intriguingly, it is the aminoterminal one third of the Sox protein that is responsible for the differential activities of Sox8 and Sox10, rather than the carboxyterminal two thirds that contain the known transactivation domains. Our study aims to provide an understanding of the relationship of Sox8 and Sox10 as paralogous transcription factors and their degree of functional redundancy in oligodendroglial cells, along with implications for health and disease.

## 1. Introduction

The SRY-box (Sox) family of transcription factors consists of 20 different members in mammals, and can be subdivided into groups A–H (SoxA–SoxH) [1]. All Sox proteins contain a high-mobility-group (HMG) domain, with similarity to the HMG domain of the male-sex-determining factor Sry, as their DNA-binding domain [2,3]. While first linked to sex determination at the time of discovery approximately 35 years ago, Sox factors are involved in numerous developmental processes, and cause several pathologies upon mutation or inactivation, including neurodevelopmental disorders [2,3,4,5]. Groups of the Sox family often contain two to three highly related Sox proteins that have emerged from genome duplication events and constitute paralogs [1,3].

The three mammalian transcription factors Sox8, Sox9 and Sox10 are nearly identical in amino acid sequence within their DNA-binding HMG domain, and are jointly classified as group E of the Sox protein family (SoxE) [3]. Whereas Sox9 and Sox10 are key regulators of ontogenetic development, influencing important specification and differentiation events, and determining cell identity during skeletal, gonadal and nervous system development, Sox8 appears expendable for the key developmental decisions in mammals, as illustrated by the mild defects in Sox8-deficient mice [6,7,8].

Frequently, two or even all three SoxE proteins are co-expressed in a particular cell type, and contribute to a common function [3]. Sox8, for instance, is expressed with Sox9 or Sox10 in Sertoli cells, chondrocytes, melanocytes, enteric glia or oligodendrocytes [8]. In all cases, the other SoxE protein is the dominant factor during development or for the maintenance of the differentiated phenotype [9,10,11,12].

In oligodendrocytes, as the myelin-forming cells of the central nervous system (CNS) that ensure saltatory conduction and rapid information processing, Sox8 and Sox10 are jointly expressed at all times of development and in the mature functional state, while Sox9 is restricted in its expression to oligodendrocyte progenitor cells (OPCs) [12,13,14]. Whereas Sox10 and Sox9 are strong determinants of OPC survival and identity, and Sox10 continues to be an essential regulator of terminal differentiation, myelin formation and myelin maintenance, Sox8-dependent effects are mild, and become mainly apparent in differentiating and adult oligodendrocytes under conditions of reduced Sox10 levels [12,15,16].

While the different impacts of Sox8 and Sox10 on the various stages of oligodendrocyte development and their function as the myelin-forming cells of the CNS are well documented, the molecular basis for this difference is still a matter of speculation. The aim of the current study was to elucidate the molecular properties and mechanisms that underlie the different levels of activity of Sox8 and Sox10 in oligodendrocytes. This was achieved through gene replacement studies conducted in the oligodendroglial Oln93 cell line, in combination with functional assays.

## 2. Results

### 2.1. Generation of Oligodendroglial Cell Lines with Selective Expression of Sox8, Sox9 or Sox10

**Figure 1:**
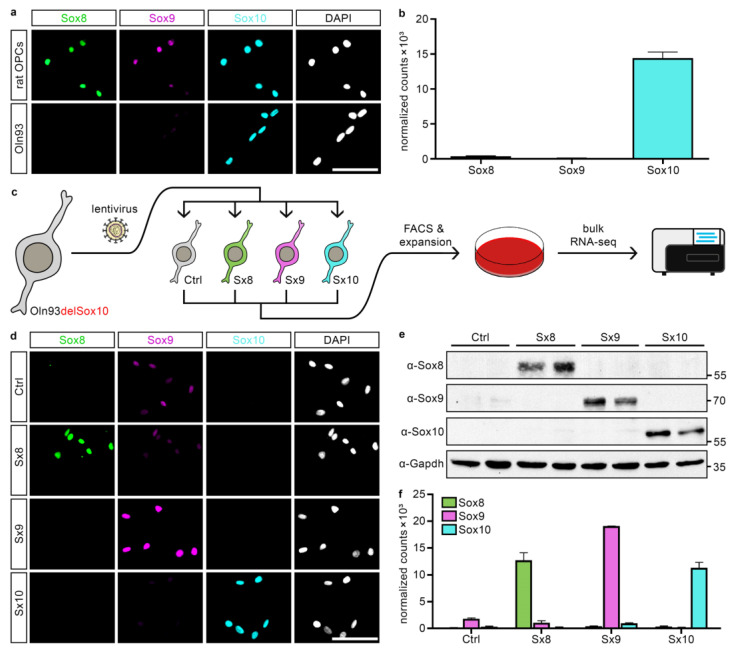
Oln93 cell lines with differential SoxE expression. (**a**) Immunocytochemical stainings of cultured rat OPCs and the original Oln93 cells with specific antibodies for Sox8, Sox9 and Sox10. Nuclei were counterstained with 4′,6-diamidino-2-phenylindole dihydrochloride (DAPI). Scale bar (valid for all panels): 50 µm; magnification: 200-fold. (**b**) SoxE transcript levels in the original Oln93 cell line according to published RNA-sequencing data (GSE136659) [17], presented as DESeq2-normalized counts. (**c**) Scheme for the generation of polyclonal cell lines that are based on a Sox10-deficient Oln93 cell clone (Oln93delSox10) and ectopically express GFP (Ctrl) and one of the three SoxE proteins (Sx8, Sx9, Sx10) after lentiviral transduction and fluorescence-activated cell sorting (FACS). (**d**) Immunocytochemical stainings of one polyclonal Oln93-derived Ctrl, Sx8, Sx9 and Sx10 line with specific antibodies for Sox8, Sox9 and Sox10. Nuclei were counterstained with DAPI. Scale bar (valid for all panels): 50 µm; magnification: 200-fold. (**e**) Western blot on extracts from two polyclonal Oln93-derived Ctrl, Sx8, Sx9 and Sx10 lines using antibodies directed against Sox8, Sox9, Sox10 or Gapdh. Numbers on the right indicate molecular weight in kilodaltons of co-electrophoresed marker proteins. (**f**) Transcript levels for mouse SoxE factors in the polyclonal Oln93-derived Ctrl, Sx8, Sx9, and Sx10 lines, according to RNA-sequencing (GSE280389), presented as DESeq2-normalized counts (n = 2).

Proliferating OPCs express Sox10 at high levels, as well as Sox8 and Sox9 at moderate levels, during their development in vivo and in vitro (Figure 1a). Despite the fact that the rat oligodendroglial cell line Oln93 is a cell culture model for OPCs, Oln93 cells express only high levels of Sox10, but virtually no Sox8 and Sox9 on both protein and transcript levels (Figure 1a,b).

To compare the roles of the three SoxE proteins in oligodendroglial cells, we made use of an Oln93 cell clone that was previously generated by CRISPR/Cas9-guided genome editing and genetically modified to be devoid of Sox10 protein (clone A in [14]). These cells (henceforth referred to as Oln93delSox10) were transduced with lentiviruses that carried GFP alone or in combination with coding sequences for one of the three mouse SoxE proteins (Figure 1c). Using fluorescence-activated cell sorting for GFP, transduced cells were collected and propagated in culture as polyclonal lines (referred to as Ctrl, Sx8, Sx9 and Sx10 lines, two of each). Immunocytochemical stainings confirmed high levels of expression of the lentivirally encoded SoxE protein in the respective cell lines (Figure 1d). When protein extracts were prepared from Ctrl, Sx8, Sx9 and Sx10 and submitted to Western blotting, we again detected high and selective expression of one of the three SoxE proteins in the polyclonal cell lines that were transduced with the corresponding lentivirus (Figure 1e). Both immunocytochemical stainings and Western blotting showed roughly comparable SoxE expression levels between the various lines (Figure 1d,e).

### 2.2. Global Impact of Sox8, Sox9 or Sox10 on the Oligodendroglial Expression Profile

To determine the impact of each SoxE protein on the Oln93 gene expression profile, we prepared the total RNA from two polyclonal Ctrl, Sx8, Sx9 and Sx10 lines each, and performed bulk RNA-sequencing. The normalized read counts for mouse *Sox8* transcripts in the Sx8 lines, *Sox9* transcripts in the Sx9 lines and *Sox10* transcripts in the Sx10 lines are roughly comparable (Figure 1f).

**Figure 2:**
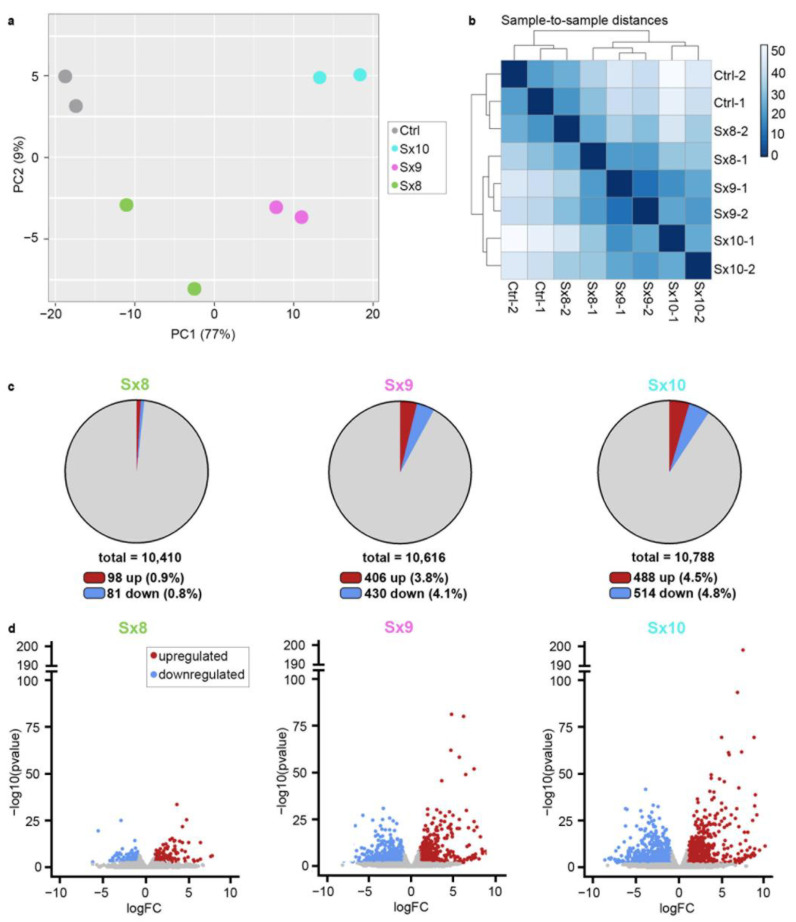
SoxE-induced expression changes in Oln93delSox10 cells. (**a**,**b**) PCA (**a**) and DESeq2 (**b**) plots comparing overall expression patterns in Oln93-derived Ctrl, Sx8, Sx9 and Sx10 cell lines, according to RNA-sequencing results. (**c**) Pie charts summarizing the fraction of upregulated (red) or downregulated (blue) differentially expressed genes (DEGs, defined by log2-fold change ≥±1, adjusted *p*-value ≤ 0.05) in Sx8, Sx9 and Sx10 cell lines compared to Ctrl. Exact numbers of DEGs and total detected genes are given below each pie chart. (**d**) Volcano plots depicting the overall distribution of DEGs in Sx8, Sx9 and Sx10 cell lines according to log2-fold change (logFC, x-axis) and *p*-value (shown as a negative decimal logarithm, y-axis).

Principal component analysis (PCA) and DESeq2 plots revealed that the cell lines that expressed the same Sox protein had the most similar expression profiles, and were more closely related to each other than to the other cell lines (Figure 2a,b). The expression profiles from the Sx8 lines were more similar to the Ctrl lines than the Sx9 or Sx10 lines. Applying a log2-fold change of ≥±1 and an adjusted *p*-value of ≤0.05, we detected 179 deregulated genes (DEGs) in Sx8 cells compared to controls, of which 98 were upregulated and 81 downregulated (Figure 2c). For Sox9 and Sox10, the number of DEGs was substantially higher, and amounted to 836 for Sox9 and 1002 for Sox10 (Figure 2c). For both Sox9 and Sox10, the number of up- and downregulated genes were balanced almost evenly. Volcano plots additionally showed that DEGs with very strong changes in expression level (log2-fold change ≥ 5) or particularly high confidence (–log10 *p*-values of ≥50) were largely restricted to the Sx9 and Sx10 lines (Figure 2d).

**Figure 3:**
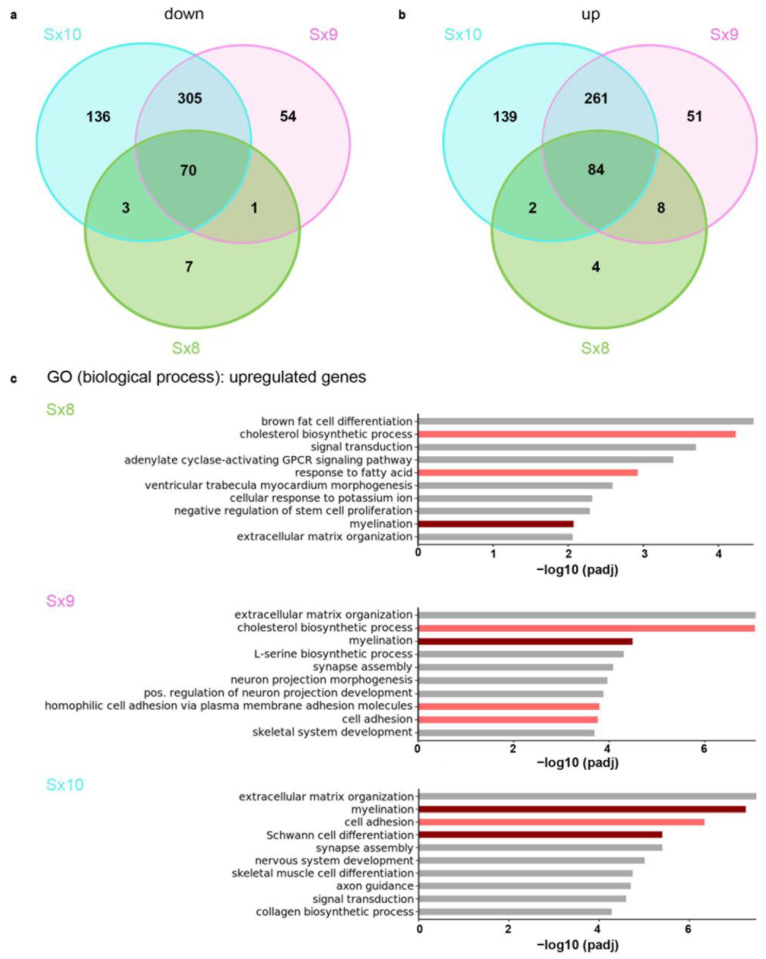
Comparison of SoxE-induced expression changes in Oln93delSox10 cells. (**a**,**b**) Venn diagrams showing the overlap of downregulated (**a**) and upregulated (**b**) genes in Sx8, Sx9 and Sx10 cell lines. Jointly deregulated genes were defined as genes similarly up- or downregulated in pairs or in all three SoxE proteins, with an adjusted *p*-value ≤ 0.05 and achieving a log2-fold deregulation of ≥±1. (**c**) Gene ontology (GO) studies on upregulated DEGs in Sx8, Sx9 and Sx10 cell lines. The top 10 terms according to adjusted *p*-value (padj) are listed. Terms related to myelinating glia are labeled in dark red; others with relevance for these cells are labeled in light red.

When the identities of the DEGs were compared between the Sx8, Sx9 and Sx10 lines, we found that 70 of the 81 downregulated genes in the Sx8 cells were also downregulated in the Sx9 and Sx10 cells (Figure 3a). For the 98 DEGs upregulated in the Sx8 cells, 84 were also upregulated in the Sx9 and Sx10 lines (Figure 3b). This amounts to percentages of 86.4% and 85.7%, respectively. There was also a very high overlap of downregulated and upregulated DEGs between the Sx9 and Sx10 lines. In total, 375 genes (corresponding to approximately 80%) were similarly downregulated and 345 genes (corresponding to approximately 78%) were similarly upregulated (Figure 3a,b).

Considering the fact that SoxE proteins act primarily as transcriptional activators, we focused on the upregulated genes for gene ontology studies (Figure 3c). These revealed that the upregulated genes exhibited a strong association with myelination and glial differentiation, but also with cholesterol and fatty acid metabolism, extracellular matrix organization and adhesion. These processes are generally associated with SoxE protein function. Lipid metabolism and adhesion are also highly relevant in both types of myelinating glia, oligodendrocytes and Schwann cells. All three SoxE proteins regulated the same biological processes, despite their different transcriptional strengths.

**Figure 4:**
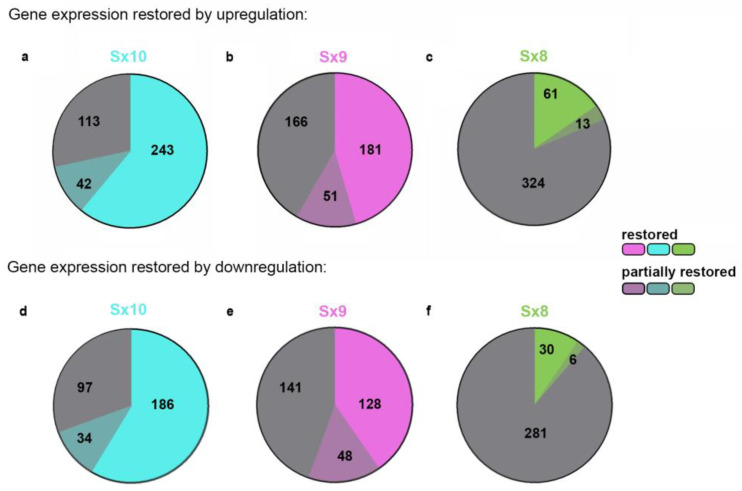
SoxE protein capability to rescue Sox10 loss in Oln93 cells. (**a**–**f**) Pie charts showing how many of the genes that were altered in their expression in Oln93 cells upon Sox10 loss (log2-foldchange of ≥±1 and adjusted *p*-value ≤ 0.05; acc. to GSE136659) were restored to their original expression in the Sx10 (**a**,**d**), Sx9 (**b**,**e**) and Sx8 (**c**,**f**) cell lines (acc. to GSE280389). Panels (**a**–**c**) focus on the 398 genes downregulated after Sox10 loss in Oln93 cells; panels (**d**–**f**) focus on the 317 upregulated genes. Shown are the number of genes whose expression was fully restored (light color) or partially restored (dark color) to their original levels prior to Sox10 loss. The gray sector in each chart represents the fraction of genes whose expression levels remained unchanged. Genes were considered restored in their expression when a log2-fold change of ≥±1 and an adjusted *p*-value ≤ 0.05 were observed in the Sx10, Sx9 and Sx8 cell lines. For a partial restoration, a log2-fold change of >0 and an adjusted *p*-value ≤ 0.05 had to be reached.

When Sox10 was originally deleted from Oln93 cells, 398 genes were downregulated, with a log2-fold change of ≥1 and an adjusted *p*-value of ≤0.05 [17]. Of these genes, 243 (corresponding to 61%) were again upregulated in the Sx10 line, with a log2-fold change of ≥1 and an adjusted *p*-value of ≤0.05, and were thus restored in their expression (Figure 4a). For the Sx9 line, the number of genes that were restored in their expression by upregulation amounted to 181 (corresponding to 45%; Figure 4b), and for Sox8, this amounted to 61 (corresponding to 15%, Figure 4c).

When we applied less stringent criteria, and asked, which of the 398 genes that were originally downregulated in Oln93 cells after Sox10 loss were upregulated in the SoxE-overexpressing Oln93Sox10del-derived polyclonal lines, with an adjusted *p*-value of ≤0.05 but without the threshold for the log2-fold change (referred to as partially restored genes), the numbers increased by 42, to a total of 285 genes (corresponding to 72%; Figure 4a), for the Sx10 line; by 51, to 232 genes (corresponding to 58%; Figure 4b), for the Sx9 line; and by 13, to 74 genes (corresponding to 19%, Figure 4c), for the Sx8 line.

The results were similar when the same analysis was performed on the 317 genes originally upregulated following Sox10 deletion in Oln93 cells with a log2-fold change of ≥1 and an adjusted *p*-value of ≤0.05. In the Sx10 line, we obtained a restoration rate of 59% (partial restoration rate of 69%), and in the Sx9 line, we obtained a restoration rate of 40% (partial restoration rate of 56%) (Figure 4d,e). In contrast, only 9% of the genes with a higher expression in Oln93delSox10 cells than in Oln93 cells were restored in their expression levels in the Sx8 line; 11% were at least partially restored (Figure 4f). Sox10 and Sox9 were more efficient than Sox8 in restoring the expression of genes originally deregulated after Sox10 deletion in the Oln93 cell line.

### 2.3. Transcriptional Activity of Sox10 and Sox8 on Oligodendroglial Regulatory Regions

**Figure 5:**
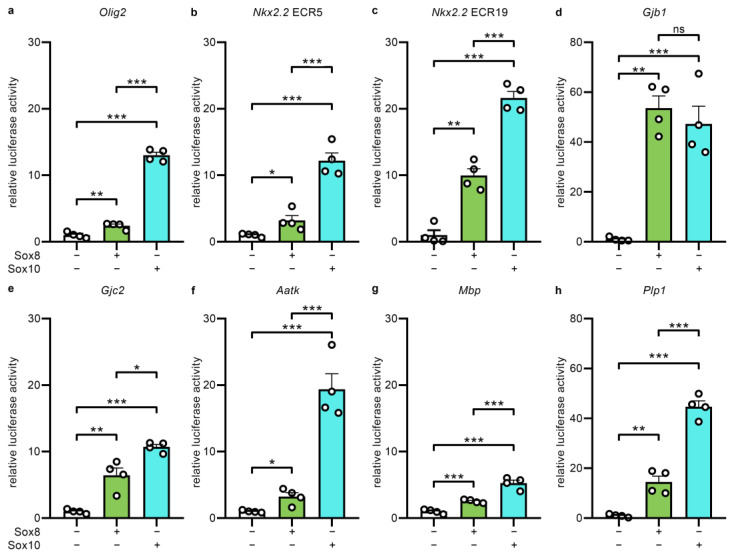
Transcriptional activity of Sox8 and Sox10 on oligodendroglial regulatory regions. (**a**–**h**) Luciferase assays in Neuro2a cells transiently transfected with reporter genes under control of the OLEa enhancer of the *Olig2* gene (**a**), the ECR5 (**b**) and ECR19 (**c**) enhancers of the *Nkx2.2* gene, the *Gjb1* (**d**) and *Gjc2* (**e**) promoters as well as the *Aatk* (**f**) enhancer, the −17 kb upstream enhancer of the *Mbp* gene (**g**) and the enhancer from the first intron of the *Plp1* gene (**h**) in the absence (−) or presence (+) of Sox10 or Sox8, as indicated below the bars. Effector-dependent activation rates are presented as fold inductions ± SEM of luciferase activity, with transfections in the absence of any Sox protein set to 1 (n = 4). Differences were statistically significant, as determined by unpaired two-tailed Student’s *t* test (ns, not significant; * *p* ≤ 0.05; ** *p* ≤ 0.01; *** *p* ≤ 0.001).

To determine the underlying cause for the differing activities of Sox10 and Sox8 in oligodendroglial cells, we aimed to reproduce these differences in reporter gene assays. In these assays, luciferase expression was under the control of Sox10-dependent regulatory regions from genes relevant for oligodendrocyte development or myelin formation and maintenance. This included previously characterized regulatory regions from the *Olig2* and *Nkx2.2* genes, the *Gjb1* and *Gjc2* connexin genes, the *Aatk* gene or the myelin genes *Mbp* and *Plp1* [17,18]. Using transiently transfected Neuro2a cells, we tested eight regulatory regions. For all of them, we were able to reproduce the previously reported activation by Sox10 (Figure 5a–h). All regions were also activated by Sox8. The Sox8-dependent activation was lowest for the OLEa enhancer from the *Olig2* gene, the ECR5 enhancer from the *Nkx2.2* gene and the enhancers of the *Aatk* and *Mbp* genes (Figure 5a,b,f,g), and it was highest for the *Gjb1* promoter (Figure 5d). With exception of the *Gjb1* promoter, Sox8-dependent activation rates were lower than the ones obtained by Sox10 in a side-by-side comparison. For some regulatory regions, the difference between Sox8 and Sox10 was substantial, such as for the *Olig2* OLEa, the *Nkx2.2* ECR5, the *Aatk* and the intronic *Plp1* enhancers (Figure 5a,b,f,h), whereas it was less pronounced for others, such as the *Nkx2.2* ECR19 and 17kb *Mbp* enhancers, and the *Gjc2* promoter (Figure 5c,e,g).

### 2.4. Genomic Binding of Sox10 and Sox8 to Oligodendroglial Regulatory Regions

**Figure 6:**
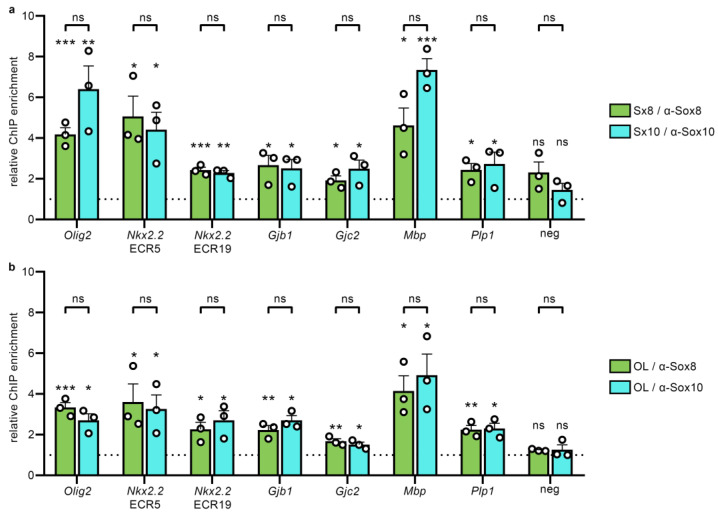
Binding of Sox8 and Sox10 to regulatory regions in oligodendroglial cells. (**a**,**b**) Immunoprecipitation experiments on chromatin of Oln93-derived Sx10 and Sx8 cell lines (**a**) as well as on chromatin of primary rat oligodendroglial cells (**b**) kept for three days under differentiating conditions, using Sox10 and Sox8 antisera, as well as the corresponding pre-immune sera, to detect occupancy of Sox10 and Sox8 on regulatory regions of the *Olig2*, *Nkx2.2* (ECR5, ECR19), *Gjb1*, *Gjc2*, *Mbp* and *Plp1* genes, and a negative control region from the *Nkx2.2* gene. Amounts of antiserum-precipitated chromatin were normalized to chromatin precipitated with the corresponding pre-immune serum, to yield relative chromatin immunoprecipitation (ChIP) enrichment rates over pre-immune serum (set to 1, indicated by the dotted line). Data represent mean values (n = 3) ± SEM, and were statistically analyzed by unpaired two-tailed Student’s *t* test (ns, not significant; * *p* ≤ 0.05, ** *p* ≤ 0.01, *** *p* ≤ 0.001).

To assess whether the differences in transcriptional activity were due to differences in the ability of Sox8 and Sox10 to bind to regulatory regions in their genomic context, we precipitated oligodendroglial regulatory regions from sheared chromatin of the Oln93-derived Sx8 and Sx10 cell lines using antibodies directed against the respective SoxE protein, and determined the enrichment in the antiserum precipitates over the pre-immune serum precipitates by quantitative PCR. For all the tested regulatory regions, we observed a statistically significant enrichment (Figure 6a). No such enrichment was obtained for a negative control region from the *Nkx2.2* gene locus. Interestingly, we could not detect any statistically significant difference in the rate of enrichment between precipitations with Sox10 antibodies in the Sx10 line and Sox8 antibodies in the Sx8 line. When solely present in Oln93 cells, Sox10 and Sox8 bound comparably well to relevant oligodendroglial regulatory regions.

In vivo, however, oligodendroglial cells express Sox10 and Sox8 simultaneously. To analyze the genomic binding of Sox10 and Sox8 in the presence of both proteins, we prepared primary cultures of oligodendroglial cells from newborn rats, and repeated immunoprecipitations on chromatin from these cells after three days under differentiating conditions. Again, we were able to detect binding of Sox10 as well as Sox8 on all of the tested regulatory regions (Figure 6b). The enrichment of regulatory regions by Sox10 or Sox8 antibodies over pre-immune serum was furthermore comparable in each case. There was no indication of a lower DNA-binding activity of Sox8 as compared to Sox10.

### 2.5. Influence of Transactivation Domains on the Transcriptional Activity of Sox10 and Sox8 in Oligodendroglial Cells

**Figure 7:**
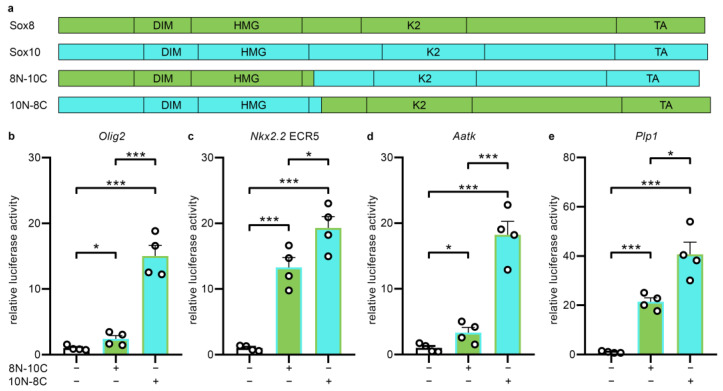
Impact of the transactivation domains on the transcriptional activity of Sox10 and Sox8 on oligodendroglial regulatory regions. (**a**) Schematic representation of the domain structure of Sox8 and Sox10 and the generated chimeras. DIM, dimerization domain; HMG, high-mobility-group domain; K2 and TA, transactivation domains. (**b**–**e**) Luciferase assays in Neuro2a cells transiently transfected with reporter genes, under control of the OLEa enhancer of the *Olig2* gene (**b**), the ECR5 enhancer of the *Nkx2.2* gene (**c**), the *Aatk* enhancer (**d**) and the intronic *Plp1* enhancer (**e**), in the absence of effector (−) or presence (+) of the Sox10/Sox8 chimeras, as indicated below the bars. Effector-dependent activation rates are presented as fold inductions ± SEM of luciferase activity, with transfections in the absence of effector set to 1 (n ≥ 4). Differences were statistically significant, as determined by unpaired two-tailed Student’s *t* test (* *p* ≤ 0.05; *** *p* ≤ 0.001).

To address the impact of the transactivation domains on the differential transcriptional activity of Sox8 and Sox10 in oligodendroglial cells, we generated chimeras, in which the aminoterminal one third of one Sox protein was combined with the carboxyterminal two thirds of the other (Figure 7a). In addition to the poorly conserved first part, the aminoterminal one third contains the highly conserved dimerization domain and the DNA-binding HMG domain (DIM and HMG in Figure 7a), whereas the carboxyterminal two thirds contain two transactivation domains (K2 and TA in Figure 7a). The resulting chimeras were then transfected in Neuro2a cells, and tested in reporter gene assays for their ability to activate the oligodendroglial gene regulatory regions with the most differential response to Sox10 versus Sox8 (Figure 5a,b,f,h). These were the *Olig2* OLEa, the *Nkx2.2* ECR5, the *Aatk* and the intronic *Plp1* enhancers. Intriguingly, we found that the chimera containing the aminoterminal one third of Sox10 and the carboxyterminal two thirds of Sox8 (10N-8C) was reproducibly more active than the chimera containing the aminoterminal one third of Sox8 and the carboxyterminal two thirds of Sox10 (8N-10C) (Figure 7b-e). The chimera containing the aminoterminal part of Sox10 and the carboxyterminal part of Sox8 behaved like Sox10 in reporter gene assays, whereas the chimera with the reciprocal design displayed Sox8-like activity (compare Figure 7b–e to Figure 5a,b,f,h).

## 3. Discussion

Many Sox proteins are crucial regulators of neurodevelopment, and mutations in the corresponding genes lead to neurodevelopmental disorders. Therefore, it is important to understand their mode of action in cells of the nervous system [5,13]. This includes *SOX2* (OMIM #184429), in which mutations lead to syndromic microphthalmia with optic nerve hypoplasia and abnormalities of the CNS (OMIM #206900). *SOX3* (OMIM #313430) mutations are a cause of X-linked intellectual developmental disorder with panhypopituitarism (OMIM #300123). Additionally, *SOX5* (OMIM #604975) and *SOX6* (OMIM #607257) are linked to Lamb–Shaffer syndrome (OMIM #616803) and Tolchin–Le Caignec syndrome (OMIM #618971), respectively [19,20,21]. Heterozygous mutations in *SOX4* (OMIM #184430) are connected to Coffin–Siris syndrome 10 (intellectual developmental disorder with speech delay and dysmorphic facies, OMIM #618506), those in *SOX11* (OMIM #600898) to Coffin–Siris syndrome 9 (intellectual developmental disorder with microcephaly and with or without ocular malformations or hypogonadotropic hypogonadism, OMIM #615866) and the ones in *SOX12* (OMIM #601947) to generalized epilepsy, intellectual disability, and childhood emotional behavioral disorders [22,23,24]. Furthermore, heterozygous *SOX10* (OMIM #602229) mutations can lead to peripheral demyelinating neuropathy, central dysmyelination, Waardenburg syndrome and Hirschsprung disease (OMIM #609136) [25,26]. Peripheral demyelinating neuropathy and central dysmyelination, as symptoms in patients with *SOX10* mutations, underscore the relevance of SOX10 for myelinating glia, including oligodendroglial cells.

Apart from Sox10, the other two SoxE proteins Sox8 and Sox9 are also expressed in oligodendroglial cells of the CNS [13]. Previous work has found Sox9 to be essential for lineage commitment and specification, and for contributing to OPC properties, whereas Sox10 is proven to be essential for oligodendroglial identity, shares functions with Sox9 in OPCs to maintain their properties, and is a central regulator of terminal differentiation, myelin formation and myelin maintenance. Compared to Sox9 and Sox10, Sox8 has exhibited only a minor impact on oligodendroglial development or in mature oligodendrocytes, where its level of expression was comparable to Sox10, and much higher than Sox9 [12,15,16]. Loss of Sox8 only led to a mild and transient delay in the early phases of oligodendrocyte differentiation [12]. Stronger effects became apparent upon the additional loss of one or two alleles of Sox10 in late-myelinating or fully mature oligodendrocytes [15,16]. This led us to assume that SoxE proteins are functionally redundant in oligodendroglial cells, but that Sox8 contributes much less to the common function than Sox10.

Here, we set out to analyze the functional differences between Sox8 and Sox10 at the molecular level. We made use of Oln93 cells [27], an immortalized oligodendroglial cell line that expresses high levels of Sox10, but, in contrast to oligodendroglial cells in vivo, virtually no Sox8 and Sox9. In these Oln93 cells, we first inactivated the *Sox10* gene [14] and then transduced the Sox10-deficient cells with lentivirus that expressed Sox8, Sox9 or Sox10. Our intention was to generate Oln93-derived oligodendroglial cell lines that each expressed one of the three SoxE proteins at similar levels, and to compare their gene expression profiles with SoxE-deficient Oln93 cells, as well as with the original Oln93 cell line. After confirming their successful establishment, the various lines were subjected to RNA-sequencing. Analysis of the data showed that Sox8 altered the expression of much fewer genes than either Sox9 or Sox10. However, most of the genes that were affected by Sox8 in their expression were also regulated by Sox9 and Sox10. The magnitude of the induced expression changes was usually higher for Sox9 and Sox10 than for Sox8. Importantly, many of the genes, whose expression was changed in the lentivirally transduced cells, had also been affected when Sox10 had been originally deleted from the Oln93 cells. All of these results suggest that the loss of Sox10 in an oligodendroglial cell line can be rescued by all three SoxE proteins, but the extent of rescue varies drastically, with Sox8 being least efficient.

**Figure 8:**
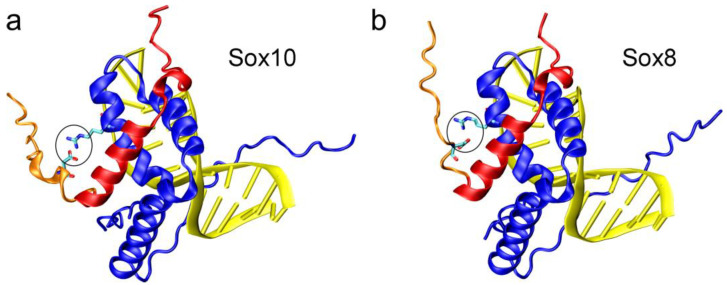
Model of the interaction between the HMG domain and dimerization domain in SoxE dimers. (**a**) Model of the Sox10 HMG domain (blue) bound to DNA (yellow) and interacting with a Sox10 dimerization domain (conserved part in red, less conserved part with residues G52-K67 in orange). The interacting residues D64 (in dimerization domain) and R120 (in HMG domain) are shown in stick presentation and are encircled. (**b**) Model of the Sox8 HMG domain (blue) bound to DNA (yellow), and interacting with a Sox8 dimerization domain (conserved part in red, less conserved part with residues G49-R60 in orange). The interacting residues D58 (in dimerization domain) and R115 (in HMG domain) are shown in stick presentation and are encircled. The different spatial arrangement of the polar residues results in a different orientation of the aminoterminus in Sox10 and Sox8.

Because all three SoxE proteins seemed to target the same genes in oligodendroglial cells, we used previously identified regulatory regions, that are active in oligodendrocytes and respond to Sox10 [17,18], to compare the transcriptional activity of Sox8 with that of Sox10. All these regions had previously been documented to be bound, in their natural context, within the oligodendroglial chromatin by Sox10, and to contain one or more functionally relevant Sox binding sites that conform to the 5′-(A/T)(A/T)CAA(A/T)G-3′ consensus binding motif [2,3,17,18]. These studies showed that Sox8 is able to activate Sox10-responsive oligodendroglial regulatory regions, but is frequently less active than Sox10. One of the reasons for a lower transcriptional activity can be a reduced ability or affinity of DNA-binding. However, using ChIP, we were unable to detect such a lower DNA-binding ability of Sox8 in the Oln93 cell lines or in primary oligodendroglial cells where Sox8 and Sox10 are co-expressed. Therefore, we have to conclude that Sox8 efficiently binds to the relevant regulatory regions in oligodendroglial cells, even in the presence of Sox10. This is not surprising, as the DNA-binding domain is very strongly conserved among all three SoxE proteins. The structures of the HMG domain and DNA-binding mode are virtually interchangeable between Sox10 and Sox8, according to in silico structure predictions (Figure 8a,b) [1,2,3].

Differences in the ability to interact with other transcription factors or co-factors, and the choice of interaction partners, are other causes for divergent transcriptional activity. This has been well documented for the activity of Sox proteins in general [28,29]. Interactions with other proteins are predominantly mediated by intrinsically disordered transactivation domains of transcription factors, which only adopt a defined structure upon interaction with other proteins. Consequently, these domains are poorly accessible to protein structure predictions [30]. All SoxE proteins carry two such transactivation domains. Both are conserved among SoxE proteins, but the degree of conservation is much lower than for the DNA-binding domain [1,2,3]. When generating chimeric proteins between Sox8 and Sox10, we had expected the transcriptional activity of the resulting chimera to correlate with the origin of its transactivation domains. Our assumption was that the transactivation domains of Sox10 would allow higher activation rates than those of Sox8. Intriguingly, this was not the case. The transcriptional activity of the chimera correlated with the origin of its aminoterminal part that consists of a poorly conserved region at the very aminoterminus, a conserved dimerization domain and the DNA-binding HMG domain.

Considering that we found neither experimental nor in silico evidence for differences in the DNA-binding activity of Sox8 and Sox10, it appears unlikely that the HMG domain was responsible for the differential activity of the two proteins because of its DNA-binding properties. The carboxyterminal part of the HMG domain is also involved in protein–protein interactions with DNA-binding domains of other transcription factors [31,32]. Therefore, we cannot exclude that differences in the interactions of the two HMG domains with partner proteins contribute to the different transcriptional activities of Sox8 and Sox10 in oligodendroglial cells. However, we consider such a scenario unlikely, because of the aforementioned near identity of the two HMG domains.

The dimerization domain of one SoxE protein interacts with the HMG domain of another SoxE protein to allow the formation of SoxE dimers. The carboxyterminal part of the dimerization domain is mainly involved in the interaction (Figure 8a,b). This part of the dimerization domain (Figure 8a,b; marked in red) is highly conserved between Sox10 and Sox8, so that the overall mode of dimerization is very similar for both SoxE proteins. Significant structural differences are only predicted for the interactions between the HMG domain and the less conserved aminoterminal part of the dimerization domain (Figure 8a,b; marked in orange, circled). These variations may affect the strength of dimerization and/or the orientation of the preceding aminoterminal region relative to the HMG domain, and may contribute to the detected differences in activity between Sox10 and Sox8. Another, and potentially more plausible, explanation is that the poorly conserved region preceding the dimerization domain (53 amino acids in Sox8 and 60 amino acids in Sox10) is responsible for the different activity of both proteins. This region is intrinsically disordered, and therefore is not amenable to modeling and meaningful structure predictions.

While our study is restricted to oligodendroglial cells, it is tempting to speculate that our findings may also be relevant in other cell types in which Sox8 is co-expressed with either Sox9 or Sox10, and appears to contribute, to a minor extent, to a common function. Examples for such cell types are enteric neural crest cells, in which Sox10 is co-expressed with Sox8 [11], as well as Sertoli cells and chondrocytes, in which Sox9 and Sox8 jointly occur [9,10,33].

As already mentioned, there are also three paralogs in other groups of the Sox family, such as the SoxB1, SoxC, SoxD and SoxF groups [1,2,3]. Co-expression and functional redundancy have also been observed for the members of these groups [34,35,36,37,38]. Among SoxC proteins, Sox4 and Sox11 furthermore appear to be generally more important than the co-expressed Sox12, whereas the overall impact of Sox5 and Sox6 on various ontogenetic processes outweighs the impact of Sox13 among the SoxD proteins. Our findings may be directly transferable to other groups of related Sox proteins, and could have broad relevance for understanding the relationships and functions of paralogous transcription factors.

## 4. Materials and Methods

### 4.1. Plasmids

Coding sequences of mouse *Sox8* (NM_011447.3), *Sox9* (NM_011448.4) and *Sox10* (NM_011437.1) were cloned into the BamHI site of pEF1a-IRES-GFP (Takara Bio, Shiga, Japan, catalog number 631971) and used to generate SoxE protein-encoding lentiviruses. pCMV5-based expression plasmids for Sox10 and Sox8 have been described [15]. pCMV5-based expression plasmids for chimeric proteins containing parts of Sox8 and Sox10 (see Figure 7a) were cloned via Gibson assembly [39], with the 2x HiFi DNA assembly master mix (New England Biolabs, Ipswich, MA, USA, catalog number E2621), using the following primers: 5′-TCAGAATTCAGATCTGGTACCATGGCCGAGGAGCAAGACCTATCAG-3′ and 5′-TACCAGAGTCTGCCTCCCCC TGGGCTGC-3′ for amino acids 1-188 of Sox10; 5′-GGGGGAGGCAGACTCTGGTACTGAACTGGGC-3′ and 5′-CTTATCGATACGCGTGGTACTCAGGGTCGGGTCA GGGT-3′ for amino acids 184-464 of Sox8; 5′-TCAGAATTCAGATCTGGTACGATGCTGGACATGAGTGAGGC-3′ and 5′-CTGGGCACTCTGAGTCGCTCCGGCCAGT-3′ for amino acids 1-183 of Sox8; and 5′-GAGCGACTCAGAGTGCCCAGGCGGGGAG-3′ and 5′-CTTATCGATACGCGTGGTACCTAAGGTCGGGATAGAGTCGTATATACTGGC-3′ for amino acids 189-466 of Sox10. Luciferase reporter plasmids containing the promoters of the *Gjb1* and *Gjc2* genes, or combinations of the β-globin minimal promoter with enhancers of the *Olig2* (OLEa), *Nkx2.2* (ECR5 and ECR19), *Aatk*, *Plp1* and *Mbp* genes, have been described and used before [17,18].

### 4.2. Cells

Rat oligodendroglial Oln93 cells (provided by C. Richter-Landsberg) [27] and mouse Neuro2a neuroblastoma cells (ATCC, CCL-131) were kept in high-glucose DMEM (Thermo Fisher Scientific, Dreieich, Germany, Gibco catalog number 11965092), supplied with 10% fetal calf serum (Anprotec, Bruckberg, Germany, catalog number AC-SM-0143) and penicillin/streptomycin (Anprotec, catalog number AC-AB-0024). In addition to the original Sox10-expressing Oln93 cells, a genome-edited Oln93 clonal line was used, in which Sox10 expression was inactivated (clone A in [14]). After lentiviral transduction and fluorescence-activated cell sorting, these Oln93delSox10 cells gave rise to the polyclonal Ctrl, Sx8, Sx9 and Sx10 lines used for RNA-sequencing (Figure 1c).

Primary rat oligodendroglial cells were obtained from newborn Wistar rats via mixed glial cultures, subsequent shake-off and microglial depletion [40,41]. Rat oligodendroglial cells were seeded on poly-ornithine-coated (Sigma-Aldrich, Hamburg, Germany, catalog number P3655) cell culture dishes, in basal medium supplemented with 10 ng/mL PDGF-AA (Thermo Fisher Scientific, PeproTech catalog number 100–13A) and 10 ng/mL bFGF (Thermo Fisher Scientific, PeproTech catalog number 100–18B). The basal medium consisted of DMEM-F12 (Thermo Fisher Scientific, Gibco catalog number 11320033) with N2 (Thermo Fisher Scientific, Gibco catalog number 17502048) and B27 supplements (Thermo Fisher Scientific, Gibco catalog number 17504044), 0.01% bovine serum albumine (Thermo Fisher Scientific, Life Technologies catalog number 15260) and penicillin/streptomycin solution. After three days in culture and a medium change on day 2, the cells were detached using accutase (Sigma-Aldrich catalog number A6964) and kept in basal medium with 10 ng/mL PDGF-AA and 10 ng/mL bFGF to maintain proliferative conditions, or transferred for 3 days to a thyroid-hormone-containing medium (Sigma-Aldrich, catalog number T6397 and T1775) to induce differentiation.

### 4.3. Immunocytochemistry

The cells were seeded and grown on poly-ornithine coated coverslips. Following fixation with 3% paraformaldehyde, the cells were first incubated with primary antisera (each at a 1:2000 dilution) directed against Sox8 (from rabbit, made in house) [11], Sox9 (from guinea pig, made in house) [14] or Sox10 (from goat, made in house) [16], and then with corresponding species-specific secondary antibodies coupled to Cy3 (Dianova, Hamburg, Germany 1:200 dilution), Cy5 (Dianova, 1:200 dilution), or Alexa Fluor 488 (Molecular Probes, Eugene, OR, USA, 1:500 dilution) fluorescent dyes. The nuclei were counterstained with 4′,6-diamidino-2-phenylindole dihydrochloride (DAPI). Fluorescent signals were documented on a camera-equipped DMI6000 B inverted microscope (Leica, Wetzlar, Germany).

### 4.4. Western Blotting

Whole cell lysates of Oln93 cells were prepared as described [18]. Proteins were size-separated on 10% polyacrylamide sodium-dodecyl-sulfate gels and blotted on nitrocellulose membranes. Immunoblotting was performed with rabbit anti-Sox8 serum (see above, 1:8000 dilution), affinity-purified rabbit anti-Sox9 serum (made in house, 1:2000 dilution) [14], rabbit anti-Sox10 serum (made in house, 1:8000 dilution) [14] or rabbit anti-Gapdh antibody (Proteintech, Rosemont, IL, USA, catalog number 10494-1-AP, 1:100,000 dilution) and protein A-HRP conjugates (ThermoFisher Scientific, Dreieich, Germany, Invitrogen catalog number 101023, 1:2000 dilution), using luminol-dependent enhanced chemiluminescence (Sigma-Aldrich, catalog number A4685).

### 4.5. RNA-Sequencing and Bioinformatics Analysis

For RNA-sequencing, total RNA was prepared from Oln93 cell lines using the RNeasy Mini Kit (Qiagen, Hilden, Germany, catalog number 74104). Contaminating DNA was removed by treatment with DNAse I, before determining quantity, purity and quality on an Agilent 5300 Fragment Analyzer (Agilent Technologies, Santa Clara, CA, USA). Libraries were prepared from 0.4 µg total RNA per sample, using the NEBNext Ultra II RNA Library Prep Kit for Illumina (New England Biolabs, Ipswich, MA, USA, catalog number E7760) according to the manufacturer’s instructions, and they underwent single-end sequencing. 50 million reads were generated, on average, per library. After investigating the quality of the raw data, sequence reads were trimmed, to remove possible adapter sequences and nucleotides with poor quality, using FASTQ Trimmer (version 1.1.5) [42]. The trimmed reads were mapped to the Rattus norvegicus genome (or the Mus musculus reference genome for specifically detecting the ectopic SoxE transcripts), available on ENSEMBL (rn5, mm10), using STAR aligner (version 2.7.11a) [43]. Unique gene hit counts were calculated by using feature counts from subread package 1.5.2 [44], and were recorded when located in exon regions. DESeq2 (version 2.11.40.8) was used for differential gene expression analysis and normalization [45]. The data were deposited in GEO, and are accessible under GSE136659 and GSE280389. GO analysis of up- and downregulated genes was performed using the Database for Annotation, Visualization and Integrated Discovery (DAVID) [46,47].

### 4.6. Reporter Gene Assays

For luciferase assays, Neuro2a cells were transfected in triplicates in 24-well plates, with 0.5 µg of luciferase reporter plasmid and 0.25 µg pCMV5-based expression plasmid, using Xfect (Takara Biotech, Kusatsu, Japan, catalog number 631318). The luciferase assay has been described in detail [18], and obtained light units were used to calculate induction rates as fold increases over luciferase activities in cells transfected with reporter and empty pCMV5.

### 4.7. Chromatin Immunoprecipitation

Oln93 cells and rat oligodendroglia, after 3 days in culture under differentiating conditions, were fixed with 1% paraformaldehyde at room temperature, before chromatin was isolated and sheared with a Bioruptor (Diagenode, Liège, Belgium) to fragments of 150-400 bp [18]. Sheared chromatin was precleared and precipitated with rabbit antisera directed against Sox10 or Sox8 (see above) and corresponding pre-immune sera. After reversal of cross-linking and proteinase K treatment (Roche Diagnostics, Penzberg, Germany, catalog number 03115844001), DNA was purified from the precipitate using the NucleoSpin Gel and PCR cleanup kit (Machery-Nagel, Düren, Germany, catalog number 740609). Quantitative PCR was performed using PowerUp SYBR Green Mastermix (Thermo Fisher Scientific, catalog number A25743) with the following primers: 5′-GTTTCCATTCTGGGGGAAAT-3′ and 5′-ACAAGTGCCTGTGGGAAGTC-3’ for *Nkx2.2* ECR 19 (rn6: chr3:141,429,475-141,429,660, size: 186 bp); 5’-CTGTCACTGGGTCCCTTTGT-3’ and 5’-CTCCATCCTCCTCCCCAATA-3’ for *Nkx2.2* ECR 5 (rn6: chr3:141,405,857-141,406,045, size: 189 bp); 5’-GGCGGGCACAGAACAGGTGG-3’ and 5’-CTCGGGGGAGCGCCTATCCT-3’ for the *Gjb1* promoter (rn6: chrX:71,277,860-71,278,152, size: 293 bp); 5’-ATCTGAATCCATGATTGTCC-3’ and 5’-GTCAGAAGTAGCCAGAGAGACTGG-3’ for the *Gjc2* promoter (rn6: chr10:45,534,672-45,534,841, size: 170 bp); 5’-GATTGCCAATTGCCTCCGAC-3’ and 5’-AGGGGGCGGTGTTTCTATTG-3’ for *Olig2* OLEa (rn6: chr11:31,357,506-31,357,820, size: 315 bp); 5′-TTTGCTCACTCGAAGGGACT-3′ and 5′-TTAGGTCCTTCTGGGGACAGT-3′ for the *Mbp* −17kb enhancer (rn6: chr18:79,388,710-79,388,944, size: 235 bp); 5′-CCAGGGCATGGGAACGAA-3′ and 5′-CTCCAAACCCTCCAAACAAGC-3′ for the intronic *Plp1* enhancer (rn6: chrX:107,501,073-107,501,260, size: 188 bp); and 5′-ACAGTCACAGCACGTGTTCC-3′ and 5′-ATGCAGGCAAAACATGTACG -3′ for a negative control region from the *Nkx2.2* locus (rn6: chr3:141,472,090-141,472,325, size: 236 bp). All the samples were processed as technical triplicates. The ΔΔCt method allowed the calculation of the percent recovery from the input. The recovery rates were used to determine the enrichment obtained with specific antisera over pre-immune sera (set to 1).

### 4.8. Structure Modeling

The interaction between the dimerization domain and the HMG domain of SoxE proteins was modeled with AlphaFold-3 [48]. The setup included residues G49-G87 of Sox8 or residues G52-G96 of Sox10 for the dimerization domain, and residues G91-S183 of Sox8 or G96-A188 of Sox10 for the HMG domain, as separate chains. In addition, a dodecameric DNA sequence (5′-CTCTTTGAGAAG-3′) was included in the modeling. Structure analysis and visualization was performed with VMD [49].

### 4.9. Quantifications and Statistical Analysis

Results from independent cell clones, experiments or separately generated samples were treated as biological replicates. The exact number of replicates is given in the respective figure legends. GraphPad Prism7 (GraphPad software, La Jolla, CA, USA) was used for statistical testing. The statistical significance of differences in luciferase activities or relative enrichment in chromatin immunoprecipitations was determined by unpaired two-tailed Student’s *t* test (*, *p* ≤ 0.05; **, *p* ≤ 0.01, ***, *p* ≤ 0.001).

## 5. Conclusions

Despite the limitations of our study, our work provides an important contribution to understanding the relation of co-expressed paralogous proteins, their respective actions and the issue of functional redundancy in general. The strong variation in the transcriptional activity of Sox8 and Sox10 in oligodendroglial cells is intriguing, and suggests that paralogous transcription factors can have a very different impact on the expression profile when co-expressed in a given cell. Differences in strength and specificities of interactions with other nuclear proteins seem to be the underlying mechanism.

The evolutionary advantage and physiological relevance of co-expressing two paralogs with markedly different transcriptional activities is not easy to explain. Although expression profiling and bioinformatic analyses, as the current state-of-the art technologies, indicate that Sox10 and Sox8 regulate more or less the same pathways and processes in oligodendroglial cells, all the available data indicate that Sox8 cannot serve as a backup of Sox10 under standard conditions [12,15,16]. For a failsafe mechanism and functional compensation, similar activities of the two paralogs would be required. Therefore, we propose that the presence of two paralogs may become useful primarily in situations where the cell encounters challenging demands, unusual signals or insults, and has to react by drastic adaptations of its gene regulatory network. In such a condition, the usually less-active paralog Sox8 may be capable of engaging in new, signal-induced functional interactions that the normally dominant paralog Sox10 is unable to make. This ability could enable Sox8 to sustain, or even enhance, transcriptional activity in challenging conditions.

In line with such an assumption, no evidence exists for a role of Sox10 in Multiple Sclerosis (MS), despite its strong influence on oligodendrocyte development and function under standard physiological conditions. Sox8, on the other hand, has been identified in two studies as a susceptibility gene for MS [50,51]. It is tempting to speculate that the usually less-active Sox8 acquires greater importance under the inflammatory conditions associated with MS, and endows the cells with capabilities to deal with such challenges. We therefore argue that knowledge and understanding of the differences between the molecular modes of action of Sox8 and Sox10 will help to lay out their respective roles not only in developmental myelination and oligodendrocyte physiology, but also in demyelinating diseases and during remyelination. This may eventually be helpful for judging and exploiting the therapeutic potential of these proteins.

## Data Availability

All the data generated or analyzed during this study are included in this published article, or were deposited in GEO under accession numbers GSE136659 and GSE280389.

## References

[1] Schepers G.E., Taesdale R.D., Koopman P. (2002). Twenty pairs of Sox: Extent, homology, and nomenclature of the mouse and human Sox transcription factor families. Dev. Cell.

[2] Kamachi Y., Kondoh H. (2013). Sox proteins: Regulators of cell fate specification and differentiation. Development.

[3] Wegner M. (1999). From head to toes: The multiple facets of Sox proteins. Nucleic Acids Res..

[4] Koopman P. (2001). Sry, Sox9 and mammalian sex determination. EXS.

[5] Angelozzi M., Lefebvre V. (2019). SOXopathies: Growing Family of Developmental Disorders Due to SOX Mutations. Trends Genet..

[6] Ming Z., Vining B., Bagheri-Fam S., Harley V. (2022). SOX9 in organogenesis: Shared and unique transcriptional functions. Cell. Mol. Life Sci..

[7] Pingault V., Zerad L., Bertani-Torres W., Bondurand N. (2022). SOX10: 20 years of phenotypic plurality and current understanding of its developmental function. J. Med. Genet..

[8] Sock E., Schmidt K., Hermans-Borgmeyer I., Bösl M.R., Wegner M. (2001). Idiopathic weight reduction in mice deficient in the high-mobility-group transcription factor Sox8. Mol. Cell. Biol..

[9] Barrionuevo F., Scherer G. (2010). SOX E genes: SOX9 and SOX8 in mammalian testis development. Int. J. Biochem. Cell Biol..

[10] Molin A.N., Contentin R., Angelozzi M., Karvande A., Kc R., Haseeb A., Voskamp C., de Charleroy C., Lefebvre V. (2024). Skeletal growth is enhanced by a shared role for SOX8 and SOX9 in promoting reserve chondrocyte commitment to columnar proliferation. Proc. Natl. Acad. Sci. USA.

[11] Maka M., Stolt C.C., Wegner M. (2005). Identification of Sox8 as a modifier gene in a mouse model of Hirschsprung disease reveals underlying molecular defect. Dev. Biol..

[12] Stolt C.C., Lommes P., Friedrich R.P., Wegner M. (2004). Transcription factors Sox8 and Sox10 perform non-equivalent roles during oligodendrocyte development despite functional redundancy. Development.

[13] Weider M., Wegner M. (2017). SoxE factors: Transcriptional regulators of neural differentiation and nervous system development. Semin. Cell Dev. Biol..

[14] Reiprich S., Cantone M., Weider M., Baroti T., Wittstatt J., Schmitt C., Kuspert M., Vera J., Wegner M. (2017). Transcription factor Sox10 regulates oligodendroglial Sox9 levels via microRNAs. Glia.

[15] Turnescu T., Arter J., Reiprich S., Tamm E.R., Waisman A., Wegner M. (2018). Sox8 and Sox10 jointly maintain myelin gene expression in oligodendrocytes. Glia.

[16] Jörg L.M., Schlötzer-Schrehardt U., Lefebvre V., Sock E., Wegner M. (2024). Transcription Factors Sox8 and Sox10 Contribute with Different Importance to the Maintenance of Mature Oligodendrocytes. Int. J. Mol. Sci..

[17] Aprato J., Sock E., Weider M., Elsesser O., Frob F., Wegner M. (2020). Myrf guides target gene selection of transcription factor Sox10 during oligodendroglial development. Nucleic Acids Res..

[18] Weider M., Starost L.J., Groll K., Kuspert M., Sock E., Wedel M., Frob F., Schmitt C., Baroti T., Hartwig A.C. (2018). Nfat/calcineurin signaling promotes oligodendrocyte differentiation and myelination by transcription factor network tuning. Nat. Commun..

[19] Hamel B.C., Smits A.P., Otten B.J., van den Helm B., Ropers H.H., Mariman E.C. (1996). Familial X-linked mental retardation and isolated growth hormone deficiency: Clinical and molecular findings. Am. J. Med. Genet..

[20] Lamb A.N., Rosenfeld J.A., Neill N.J., Talkowski M.E., Blumenthal I., Girirajan S., Keelean-Fuller D., Fan Z., Pouncey J., Stevens C. (2012). Haploinsufficiency of SOX5 at 12p12.1 is associated with developmental delays with prominent language delay, behavior problems, and mild dysmorphic features. Hum. Mutat..

[21] Tolchin D., Yeager J.P., Prasad P., Dorrani N., Russi A.S., Martinez-Agosto J.A., Haseeb A., Angelozzi M., Santen G.W.E., Ruivenkamp C. (2020). De Novo SOX6 Variants Cause a Neurodevelopmental Syndrome Associated with ADHD, Craniosynostosis, and Osteochondromas. Am. J. Hum. Genet..

[22] Tsurusaki Y., Koshimizu E., Ohashi H., Phadke S., Kou I., Shiina M., Suzuki T., Okamoto N., Imamura S., Yamashita M. (2014). De novo SOX11 mutations cause Coffin-Siris syndrome. Nat. Commun..

[23] Zawerton A., Yao B., Yeager J.P., Pippucci T., Haseeb A., Smith J.D., Wischmann L., Kuhl S.J., Dean J.C.S., Pilz D.T. (2019). De Novo SOX4 Variants Cause a Neurodevelopmental Disease Associated with Mild Dysmorphism. Am. J. Hum. Genet..

[24] Treccarichi S., Cali F., Vinci M., Ragalmuto A., Musumeci A., Federico C., Costanza C., Bottitta M., Greco D., Saccone S. (2024). Implications of a De Novo Variant in the SOX12 Gene in a Patient with Generalized Epilepsy, Intellectual Disability, and Childhood Emotional Behavioral Disorders. Curr. Issues Mol. Biol..

[25] Inoue K., Tanabe Y., Lupski J.R. (1999). Myelin deficiencies in both the central and peripheral nervous system associated with a SOX10 mutation. Ann. Neurol..

[26] Pingault V., Bondurand N., Kuhlbrodt K., Goerich D.E., Prehu M.-O., Puliti A., Herbarth B., Hermans-Borgmeyer I., Legius E., Matthijs G. (1998). Sox10 mutations in patients with Waardenburg-Hirschsprung disease. Nat. Genet..

[27] Richter-Landsberg C., Heinrich M. (1996). OLN-93: A new permanent oligodendroglia cell line derived from primary rat brain glial cultures. J. Neurosci. Res..

[28] Kamachi Y., Uchikawa M., Kondoh H. (2000). Pairing SOX off: With partners in the regulation of embryonic development. Trends Genet..

[29] Kondoh H., Kamachi Y. (2010). SOX-partner code for cell specification: Regulatory target selection and underlying molecular mechanisms. Int. J. Biochem. Cell Biol..

[30] Udupa A., Kotha S.R., Staller M.V. (2024). Commonly asked questions about transcriptional activation domains. Curr. Opin. Struct. Biol..

[31] Remenyi A., Lins K., Nissen L.J., Reinbold R., Schöler H.R., Wilmanns M. (2003). Crystal structure of a POU/HMG/DNA ternary complex suggests differential assemby of Oct4 and Sox2 on two enhancers. Genes Dev..

[32] Werner M.H., Huth J.R., Gronenborn A.M., Clore G.M. (1995). Molecular basis of human 46X,Y sex reversal revealed from the three-dimensional solution structure of the human SRY-DNA complex. Cell.

[33] Koopman P. (2005). Sex determination: A tale of two Sox genes. Trends Genet..

[34] Bergsland M., Werme M., Malewicz M., Perlmann T., Muhr J. (2006). The establishment of neuronal properties is controlled by Sox4 and Sox11. Genes Dev..

[35] Bylund M., Andersson E., Novitch B.G., Muhr J. (2003). Vertebrate neurogenesis is counteracted by Sox1-3 activity. Nat. Neurosci..

[36] Stolt C.C., Schlierf A., Lommes P., Hillgärtner S., Werner T., Kosian T., Sock E., Kessaris N., Richardson W.D., Lefebvre V. (2006). SoxD proteins influence multiple stages of oligodendrocyte development and modulate SoxE protein function. Dev. Cell.

[37] Lefebvre V. (2019). Roles and regulation of SOX transcription factors in skeletogenesis. Curr. Top. Dev. Biol..

[38] Dy P., Penzo-Mendez A., Wang H., Pedraza C.E., Macklin W.B., Lefebvre V. (2008). The three SoxC proteins--Sox4, Sox11 and Sox12--exhibit overlapping expression patterns and molecular properties. Nucleic Acids Res..

[39] Gibson D.G., Young L., Chuang R.Y., Venter J.C., Hutchison C.A., Smith H.O. (2009). Enzymatic assembly of DNA molecules up to several hundred kilobases. Nat. Methods.

[40] McCarthy K.D., DeVellis J. (1980). Preparation of separate astroglial and oligodendroglial cell cultures from rat cerebral tissue. J. Cell. Biol..

[41] Yang J., Cheng X., Shen J., Xie B., Zhao X., Zhang Z., Cao Q., Shen Y., Qiu M. (2016). A Novel Approach for Amplification and Purification of Mouse Oligodendrocyte Progenitor Cells. Front. Cell Neurosci..

[42] Blankenberg D., Gordon A., Von Kuster G., Coraor N., Taylor J., Nekrutenko A., Galaxy T. (2010). Manipulation of FASTQ data with Galaxy. Bioinformatics.

[43] Dobin A., Davis C.A., Schlesinger F., Drenkow J., Zaleski C., Jha S., Batut P., Chaisson M., Gingeras T.R. (2013). STAR: Ultrafast universal RNA-seq aligner. Bioinformatics.

[44] Liao Y., Smyth G.K., Shi W. (2014). featureCounts: An efficient general purpose program for assigning sequence reads to genomic features. Bioinformatics.

[45] Love M.I., Huber W., Anders S. (2014). Moderated estimation of fold change and dispersion for RNA-seq data with DESeq2. Genome Biol..

[46] Sherman B.T., Hao M., Qiu J., Jiao X., Baseler M.W., Lane H.C., Imamichi T., Chang W. (2022). DAVID: A web server for functional enrichment analysis and functional annotation of gene lists (2021 update). Nucleic Acids Res..

[47] Huang D.W., Sherman B.T., Lempicki R.A. (2009). Systematic and integrative analysis of large gene lists using DAVID bioinformatics resources. Nat. Protoc..

[48] Abramson J., Adler J., Dunger J., Evans R., Green T., Pritzel A., Ronneberger O., Willmore L., Ballard A.J., Bambrick J. (2024). Accurate structure prediction of biomolecular interactions with AlphaFold 3. Nature.

[49] Humphrey W., Dalke A., Schulten K. (1996). VMD: Visual molecular dynamics. J. Mol. Graph..

[50] Lill C.M., Schjeide B.M., Graetz C., Ban M., Alcina A., Ortiz M.A., Perez J., Damotte V., Booth D., Lopez de Lapuente A. (2013). MANBA, CXCR5, SOX8, RPS6KB1 and ZBTB46 are genetic risk loci for multiple sclerosis. Brain.

[51] Sawcer S., Hellenthal G., Pirinen M., Spencer C.C., Patsopoulos N.A., Moutsianas L., Dilthey A., Su Z., Freeman C., Hunt S.E. (2011). Genetic risk and a primary role for cell-mediated immune mechanisms in multiple sclerosis. Nature.

